# Effect of Post-Grazing Sward Height, Sire Genotype and Indoor Finishing Diet on Steer Intake, Growth and Production in Grass-Based Suckler Weanling-to-Beef Systems

**DOI:** 10.3390/ani11092623

**Published:** 2021-09-07

**Authors:** Peter R. Doyle, Mark McGee, Aidan P. Moloney, Alan K. Kelly, Edward G. O’Riordan

**Affiliations:** 1 Teagasc, Animal & Grassland Research and Innovation Centre, Grange, Dunsany, C15 PW93 Co. Meath, Ireland; Mark.McGee@teagasc.ie (M.M.); aidan.moloney@teagasc.ie (A.P.M.); 2 School of Agriculture and Food Science, University College Dublin, Belfield, D04 V1W8 Dublin, Ireland; alan.kelly@ucd.ie

**Keywords:** beef cattle, breed maturity, concentrate supplementation, grass-fed beef, grazing behaviour, grazing intensity, forage-only, herbage production, pasture, residual sward height

## Abstract

**Simple Summary:**

Grass-fed beef is becoming popular; however, there is little research information on optimising beef cattle performance in such production systems. In grass-forage-only beef systems, the removal of dietary concentrates increases the difficulty in achieving target live-weight performance and carcass fatness. Post-grazing sward height can potentially influence animal live-weight gain at pasture, whilst sire breed maturity (genotype) can potentially influence carcass fatness and, therefore, the duration required to achieve a commercially acceptable carcass fat score. Therefore, contrasting post-grazing sward heights and beef steer genotypes were evaluated within a grass-forage-only and grass-forage + concentrate production system. The high post-grazing sward height (6 cm) increased intake and live-weight gain at pasture and resulted in a heavier carcass after an indoor finishing period compared to the low post-grazing sward height (4 cm). The early-maturing genotype had a greater intake, live-weight gain and carcass fatness, but similar carcass weight and lower conformation score compared to the late-maturing genotype. Although concentrate supplementation indoors increased carcass weight and fatness, grass-forage-only steers still achieved a commercially-acceptable fat score when slaughtered at 24 months of age. In conclusion, grazing higher sward residuals and utilising early-maturing animal genotypes can increase live-weight pasture gain and carcass fatness, respectively, in grass-forage beef production systems.

**Abstract:**

This study evaluated the effects of post-grazing sward height (PGSH, 4 or 6 cm) on herbage production, its nutritive value, dry matter (DM) intake, grazing behaviour and growth of early- (EM) and late-maturing (LM) breed suckler steers (*n* = 72), and the subsequent effect of indoor finishing diet (grass silage + 3.8 kg concentrate DM/head daily (SC), or grass silage only (SO)) on performance and carcass traits. Animals rotationally grazed pasture for 196 days, followed by indoor finishing for 119 days. At pasture, daily live-weight gain (LWG) was 0.10 kg greater for PGSH-6 than PGSH-4, resulting in a tendency for carcass weight to be 11 kg heavier. Although EM had a 0.10 kg greater daily LWG at pasture than LM, carcass weight did not differ between the genotypes. There was a genotype × PGSH interaction for carcass fat score, whereby there was no difference between EM-4 (8.83, 15-point scale) and EM-6 (8.17), but LM-6 (7.28) was greater than LM-4 (6.33). Although concentrate supplementation during indoor finishing increased carcass weight (+37 kg) and fat score (1.75 units), the majority of steers (83% of EM and 78% of LM) achieved a commercially-acceptable carcass fat score (6.78) at slaughter in the grass-forage-only system.

## 1. Introduction

Maximising herbage production and animal live-weight gain (LWG) at pasture, optimising stocking rate and minimising purchased concentrates are identified as key drivers of profitability in suckler beef systems [1]. Grass-based suckler beef production systems in temperate climates entail spring-calving cows rearing their own calves until weaning at the end of the grazing season [2]; these calves are usually sold for subsequent finishing. Such weanling-to-beef steer systems typically involve a post-weaning indoor ‘store’ feeding period, where animals are offered a restricted-energy diet based on grass silage and limited concentrates, which is then followed by a grazing season exploiting compensatory growth [3]. These animals finally have an indoor finishing period based on grass silage and higher levels of concentrate supplementation, after which animals are slaughtered at 24 months of age [2,4]. Although concentrates are included in those beef systems to enhance animal growth, consumer interest in the source of their food, its environmental footprint, human health impact [5] and the desire not to use human-edible feed in ruminant production systems [6], has supported the need to develop grass-forage-only production systems. However, there is little published information on producing steers on grass-forage-only production systems, where concentrates are omitted from the animal’s diet [4]. Pasture management challenges and the length of time required for animals to achieve required carcass specifications are identified as production-limiting factors in grass-forage-only production systems [7]. As fat is the last carcass component (after bone and muscle) to be deposited, attaining acceptable carcass fatness is the primary limiting component in grass-based beef systems [4]. Failure to achieve a carcass fatness of >6.0 (1–15 scale) can result in financial penalisation [8]. Consequently, strategies that enhance individual animal LWG and subcutaneous fat deposition need to be investigated in order to obtain commercially-acceptable beef carcasses by 24 months-of-age.

Post-grazing sward height (PGSH) as a grassland management tool could increase beef cattle LWG from pasture in grass-forage-only systems [9,10,11]. However, current grazing severity guidelines for rotationally stocked temperate pastures are primarily based on dairy cow studies in Ireland [12,13] and New Zealand [14]. In these grass-based dairy systems, grazing to a compressed PGSH of ca. 4 cm, which optimises herbage production [15] and nutritive supply [16], stocking rate and ultimately milk solids yield output per hectare [17,18], is recommended [18], although there is a reduction in individual cow performance [15,16,17]. This PGSH is now also recommended for beef cattle [19]; however, these guidelines may not be directly transferable to rotational grazing systems for beef cattle, as stocking rates are generally much lower than dairy farms [13,14], and therefore individual animal performance is much more important. There is evidence that grazing to a low PGSH (ca. 4 cm) in rotational grazing systems with beef cattle restricts animal growth [10,11]. Increasing PGSH from 4 to ca. 6 cm may potentially increase individual animal LWG at pasture, thereby reducing the necessity for expensive supplementary concentrate during the indoor finishing period in order to achieve an acceptable carcass fatness in beef steers. However, there is relatively little published information appraising this within weanling-to-beef suckler systems.

Late-maturing (LM) genotypes predominate in the suckler herd in Ireland [20] and many other European countries [21,22], mainly due to their superior carcass growth and conformation compared to early-maturing (EM) genotypes [23]. However, compared to LM, EM steers may be more suitable to grass-forage-only systems due to their propensity for greater fat deposition at a younger age [4], and thus potentially reducing the time to slaughter. Nonetheless, there is a dearth of published information [4] comparing the intake, growth, carcass traits and thus, ‘suitability’ of LM and EM steers within rotationally stocked, grass-forage-only weanling-to-beef suckler systems, and no published research evaluating the interaction between steer ‘maturity’ and PGSH within such systems.

Therefore, the objective of this experiment was to determine the effects of PGSH on herbage accumulation and its nutritive value, dry matter intake (DMI), grazing behaviour and growth of EM and LM breed suckler steers, and the subsequent effect of indoor finishing diet on performance and carcass traits within grass-based weanling-to-beef production systems.

## 2. Materials and Methods

**Figure 1:**
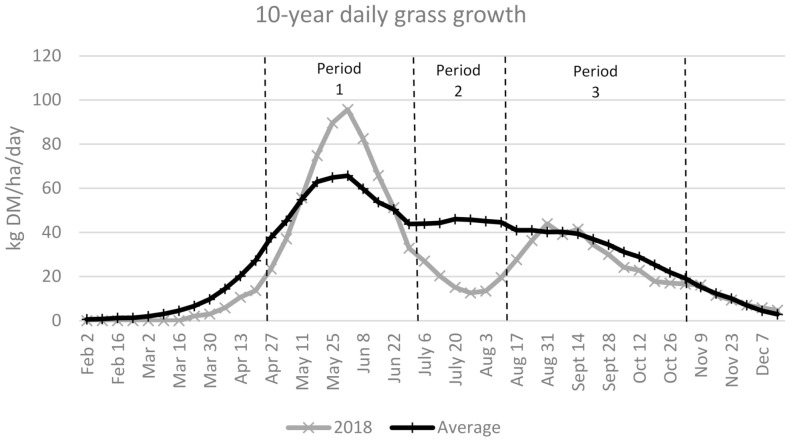
Daily grass growth for 2018 compared to the previous 10-year average and associated grazing periods implemented during the study.

This study was conducted at Teagasc, Grange Research Centre, Ireland (Longitude 6°40′ W; Latitude 53°30′ N; Elevation 92 m above sea level) between October 2017 and March 2019, on a moderately well-drained brown earth with gleying and a clay loam texture soil type. Mean rainfall and soil temperature (50 mm depth) at the centre during the 2018 grazing season (April to October) was 296 mm and 13.4 °C, respectively. This compares with the 47-year average of 484 mm and 12.7 °C, respectively. The 2018 grazing season experienced low rainfall during the months of May (31 mm), June (11 mm) and July (42 mm), compared to the long-term averages of 62, 64 and 65 mm, respectively, which reduced grass growth during this key period compared to the long-term mean (Figure 1).

Animal procedures performed in this experiment were approved by the Teagasc Animal Ethics Committee and were conducted in accordance with the European Communities (Amendment of Cruelty to Animals Act 1876) Regulation 2002 and 2005.

### 2.1. Animals

Seventy-two spring-born, EM (Aberdeen Angus and Hereford, *n* = 36) and LM (Limousin and Charolais, *n* = 36) sired, weaned suckler-bred bulls (312 ± 12.6 kg) bred from LM crossbred dams were sourced from commercial farms in Ireland and transferred to Grange Research Centre in mid-October, at ca. 232 days (7.6 months) of age. Animals were representative of the national breed population based on their estimated breeding value for Terminal Index and carcass weight, acquired by the Irish Cattle Breeding Federation (ICBF, Cork, Ireland). Following arrival at the centre, and subsequently as required, the animals were treated for internal and external parasites and vaccinated against Clostridial and respiratory diseases. Prior to commencing the experiment, the animals were castrated using a ‘burdizzo’ by a veterinarian and rotationally stocked at pasture for four weeks before housing for their first winter.

### 2.2. Experimental Design and Management

Three weeks post-arrival, steers were weighed on two consecutive days at pasture and within sire breed, blocked by live-weight and randomly assigned to one of six grazing groups. The grazing group was assigned to a two (sire genotype: EM and LM) by two (PGSH: 4 or 6 cm (compressed sward height)) factorial arrangement of treatments. Within each PGSH, there were three replicate groups per genotype, resulting in twelve grazing groups of six steers each.

Steers were accommodated indoors in slatted floor pens in their respective groups of six (lying area = 2.20 m^2^/animal). The mean age and weight at the beginning of the experiment (14 November 2017) was 262 days (8.6 months) (s.d. 27.8) and 332 kg (s.d. 24.2), respectively. For their first winter, steers were offered only grass silage (in vitro DM digestibility (DMD), 739 g/kg) ad libitum, plus a mineral and vitamin supplement for 156 days. Steers were turned out to pasture (19 April 2018), where they were rotationally stocked on *Lolium perenne* dominant swards in their breed-specific replicate grazing groups for 196 days.

At the end of the grazing season (1 November 2018), the steers were accommodated indoors in concrete slatted floor pens in their respective sub-groups (lying area = 2.68 m^2^/animal) for the finishing period. Within grazing groups, the steers were blocked on live-weight and randomly assigned to one of two indoor finishing diets, (1) grass silage (756 g/kg DMD) ad libitum supplemented with 3.8 kg concentrate DM (SC) or (2) grass silage (756 g/kg DMD) only (SO) ad libitum plus minerals and vitamins (offered at a level equivalent to that contained in the concentrates). Initially, all steers were individually offered grass silage ad libitum during an adaption and training period of 10 days to acclimatise to Calan gates (American Calan Inc., Northwood, NH, USA), following which they were offered their respective finishing diet for 119 days. At the end of the indoor finishing period (12 March 2019), the animals were slaughtered in a commercial abattoir at ca. 24 months-of-age.

### 2.3. Pasture Management

The experimental area was a permanent grassland site comprising of 24.53 ha, which was initially divided into a grazing (9.12 ha; 37%) and silage (15.41 ha; 63%) area. The global stocking rate was 2.9 steers/ha (2.1 livestock units/ha). The grazing area consisted of forty permanent paddocks, which were balanced for location within the farm and randomly assigned to four equal-sized 2.28 ha grazing area farmlets, balanced for starting herbage mass. Each grazing farmlet was assigned to one treatment and contained three replicated grazing groups. Each paddock was divided into three sub-paddocks (0.076 ha each), where the three replicated groups grazed their respective sub-paddock to the assigned PGSH. The three groups moved at the same time to the next sub-paddock and returned to their same sub-paddock on each rotation. In order to achieve the target PGSH, paddock residency time and daily area grazed varied between the two PGSH treatments. Daily herbage allowance was calculated as described by Ganche et al. [15].

Overall, pasture supply was managed independently on each farmlet using the farm cover technique as described by O’Donovan and Dillon [24]. Pasture supply was measured weekly on all paddocks using a rising plate meter whereby fifty random heights were taken across each paddock (220 heights/ha), and herbage yield (kg DM/ha) was estimated using the equation of O’Riordan et al. [25]. Herbage in excess of grazing requirements (pre-grazing herbage mass > ca. 2300 kg DM/ha) in the grazing area was cut using a rotary mower, wilted for 36 h, and harvested as silage using a precision-chop harvester and ensiled without an additive in a bunker silo covered with two layers of black polythene sheet and weighted down; herbage off-take yield was recorded (see below).

In addition, the assigned silage area was sub-divided into 16 equal-sized areas, which were randomly assigned to each treatment resulting in 3.85 ha/treatment. Silage was harvested three times (22 May, 27 June and 3 September) to provide forage for the weanlings during the first winter and steers during the finishing period. Due to insufficient rainfall, pasture growth was substantially below average in July and August (Figure 1), and pasture supply on the grazing area was insufficient to meet animal demand. Consequently, all groups were moved simultaneously to the silage area, where they grazed a similar pre-grazing herbage mass (4500 kg DM/ha) to a common PGSH of 6 cm for 43 days before together returning to their respective designated grazing areas. To account for the disruption in pasture supply, the grazing season was divided into three periods; period 1 (19 April–2 July) where the animals grazed a similar pre-grazing herbage mass to their assigned PGSH on their assigned grazing area, period 2 (2 July–10 August) where the steers grazed the silage area to a common PGSH, and period 3 (10 August–1 November) where the steers again grazed a similar pre-grazing herbage mass to their assigned PGSH. Reflecting the seasonality of herbage growth, pasture areas that were harvested for silage earlier in the year were added to the grazing area on 20 September. Apart from removing herbage surplus to grazing requirements, the paddocks were not mechanically topped. Each farmlet received chemical nitrogen in four applications totalling 152 kg nitrogen/ha on the grazing area and three applications totalling 254 kg N/ha on the silage area, averaging 216 kg N/ha across the experimental area. Phosphorus and potassium fertiliser application rates were based on soil test results.

### 2.4. Pasture Measurements

Pre- and PGSH was determined using a rising plate meter, where thirty random heights in each sub-paddock (~400 heights/ha) were recorded, and pre- and post-grazing herbage mass (kg DM/ha) (>4 cm) were estimated based on these heights using the equation of O’Riordan et al. [25]. Pre- and post-grazing herbage mass in each sub-paddock was also directly determined by cutting one 10.0 m long by 0.53 m wide representative strip of pasture, to the assigned PGSH (4 or 6 cm stubble height), with a rotary lawnmower (Honda HRX537VYEA, Honda distributors, Dublin, Ireland) and the herbage collected and weighed. A representative 200 g herbage sample from each strip was immediately dried at 90 °C for 16 h to determine DM concentration. Pre-grazing herbage mass was subsequently adjusted for herbage growth that took place between the sampling date and when steers began to graze the paddock. The herbage growth rate for any period was estimated as the difference between pre-grazing herbage mass in one cycle and the post-grazing herbage mass of the previous grazing cycle, divided by the number of days between the two yield estimates. The annual herbage accumulation on the grazing area was calculated as the sum of the differences between pre- and post-herbage mass of the same cycle or rotation. This was adjusted for herbage growth rate while animals were resident in the sub-paddock (number of residency day’s × daily herbage growth rate). Annual herbage accumulation was further sub-divided into what was grazed or removed as surplus herbage (silage) or remained as a ‘closing cover’ at the end of the grazing season from each sub-paddock. Grazing group (six steers) DMI was estimated during periods 1 and 3 by recording herbage disappearance rate [26], which was adjusted for herbage growth rate while animals were residents in the sub-paddock. Rest period, rotation (or stocking) cycle and canopy density (above the assigned PGSH) were measured as defined by Allen et al. [27]. Reduced herbage mass was calculated as pre-grazing herbage mass minus post-grazing herbage mass. Herbage utilisation was calculated above 4 cm for both treatments as described by Tuñon et al. [12].

Leaf, stem and dead proportions were calculated from three assigned base paddocks per treatment as detailed in Ganche et al. [13]. However, the DM proportions were only determined above the assigned PGSH (4 or 6 cm). Leaf, stem and dead herbage mass yields were determined by multiplying pre-grazing herbage mass (>4 or 6 cm) by morphology DM proportion.

### 2.5. Grazing Behaviour

Grazing behaviour was recorded for each animal over six consecutive days between 27 August and 16 September (day 130–150 of the grazing season) using the RumiWatch noseband sensor (Itin & Hoch GmbH, Liestal, Switzerland) [28]. The grazing behaviour data were converted into 1-h summaries with the RumiWatch converter V.0.7.4.13 (Itin & Hoch GmbH) [28]. During these measurement periods, the pasture was allocated to each grazing treatment group on a 48-h basis such that each grazing group would achieve their designated PGSH three times during the 6-day measurement period, to permit repeated measures of ingestive behaviour during the grazing process.

### 2.6. Indoor Feed Intake

Fresh silage was weighed daily and offered ad libitum (proportionately 0.1 in excess of daily intake) on a pen basis during the first winter (weanlings) and on an individual animal basis during the finishing period. Uneaten silage was weighed and discarded twice weekly during the first winter and weighed daily and discarded twice weekly during the finishing period. The concentrate (coarse mixture—862 g/kg rolled barley, 60 g/kg soyabean meal, 50 g/kg molasses and 28 g/kg minerals and vitamins) supplement was gradually increased to 4.8 kg/animal daily (3.83 kg DM) over eight days and was individually offered once daily on top of the silage. Animals on SO received 107 g/day of general-purpose mineral-vitamin supplement (calcium 25.0%, sodium 12.4%, vitamin A 500,000 IU/kg, D3 100,000 IU/kg, E 1500 mg/kg, B12 750 mg/kg and B1 250 mg/kg) on top of the silage (equivalent to that offered in the concentrates for SC). All animals had continuous access to clean, fresh water.

### 2.7. Feedstuff Analysis

Representative samples of offered grass silage and concentrates were obtained twice weekly at feeding. Duplicate 200 g samples of silage and concentrates were dried at 90˚C for 16 h for DM determination, and grass silage DM was corrected for volatile losses [29]. Sample processing and chemical analysis of grazed herbage, silage and concentrates was conducted using the methods reported by Lenehan et al. [30], as appropriate. The Unité Fourragère Viande (UFV) energy values of the concentrate and grass silage offered was estimated as described by Noziere et al. [31] and O’Mara [32], respectively. The DM, DMD, neural detergent fibre (NDF), crude protein (CP) and UFV concentration of the grass silage offered during the first winter was 235 g/kg, 739, 469, 171 g/kg DM and 0.799 kg DM, respectively. Corresponding values during the finishing period were 323 g/kg, 756, 437, 164 g/kg DM and 0.814 kg DM. The concentrate DM, NDF, neutral cellulase gammanase digestibility, CP and UFV concentration was 769 g/kg, 146, 954, 144 g/kg DM and 1.22 kg DM, respectively.

### 2.8. Animal Live-Weight and Body Composition

Live-weight was measured using a calibrated scales (Tru-Test XR3000, load bars XHD 10,000, Auckland, New Zealand) on two consecutive days at critical time points including, prior to housing for the first winter, prior to turnout to pasture (start of grazing period 1), post-turnout to pasture (to quantify gut-fill changes during the transition from a grass silage-based to grass-based diet), at the start of grazing periods 2 and 3, prior to housing, ten days post-housing for the indoor finishing period and finally on three consecutive days before slaughter. Additionally, live-weight was recorded every two weeks throughout the study. Live-weight gain was calculated as the accumulated live-weight over a specified period of time. Average daily gain (ADG) was calculated as the LWG over a specified period divided by the duration of that period.

Animals were ultrasonically scanned at turnout to pasture, at housing for the finishing period and at pre-slaughter using an automatic real-time scanner (model—ECM ExaGo Veterinary scanner, with a 3.5 MHz linear transducer, IMV imaging, Meath, Ireland) to determine *M. longissimus* and backfat depth as described by Lenehan et al. [30]. To provide a more objective description of morphology, skeletal measurements were recorded at the same time points as ultrasonic measurements [30].

### 2.9. Carcass and Post-Slaughter Measurements

Cold carcass weight was estimated as 0.98 of the hot carcass weight. The kill-out proportion was calculated as cold carcass weight expressed as a proportion of pre-slaughter live-weight. Carcasses were graded mechanically for conformation and fat score on a 15-point scale according to the EU beef carcass classification system as described by Mezgebo et al. [33]. Subcutaneous fat colour (L, a, b) on the shoulder, lumbar and rump regions on the right side of each carcass was measured (48 h post-mortem) using a Miniscan EZ portable spectrophotometer (Hunter Associates Laboratory Inc., Reston, VA, USA) as described by Mezgebo et al. [33].

### 2.10. Systems Output/Ha Measurements

The grazing area used per rotation and the area of excess herbage removed per rotation (from the grazing area only) were calculated using the same equation as Wims et al. [34]. The stocking rate was calculated as:

(1) $$\frac{\mathrm{Number}\;\mathrm{of}\;\mathrm{steers}}{\mathrm{average}\;\mathrm{grazing}\;\mathrm{area}\;\mathrm{used}\;\mathrm{per}\;\mathrm{rotation}}$$

Total LWG/ha was calculated for each treatment as:

(2) $$\frac{\sum \mathrm{LWG}\;\mathrm{per}\;\mathrm{treatment}}{\mathrm{grazing}\;\mathrm{area}\;\mathrm{used}\;\mathrm{per}\;\mathrm{rotation}}$$

Total silage demand for a weanling-to-beef steer system was calculated as the combined silage intake of the animal during the first winter and indoor finishing period (animal unit), and this was adjusted for potential silage losses at ensiling or edible silage recovered [35] as:

(3) $$\left(\frac{\sum \mathrm{Silage}\;\mathrm{DM}\;\mathrm{consumed}}{0.78}\right)\times 1$$

The silage preserved per animal unit is the sum of silage yield from the grazing and silage areas, divided by the number of animals in that treatment.

### 2.11. Statistical Analysis

All data pertaining to herbage measurements were analysed using the MIXED procedure of Statistical Analysis Software (SAS), where the experimental unit was the paddock within grazing area farmlet (*n* = 10). The data pertaining to sward characteristics, herbage accumulation and grazing rotation cycles were analysed using a statistical model, which contained the fixed effects of PGSH, genotype and their interactions. Data averaged per paddock were weighted for frequency of grazing (i.e., the number of times the paddock was defoliated). Differences between means were tested for significance using the PDIFF statement and adjusted by Tukey. For daily herbage growth rate, repeated measures were used for grazing season growth stages, where stages were considered as early (April and May), mid (June and July) and late (August, September and October) season. The number of data points in each growth stage differed. Data for herbage nutritive value were analysed using the same model, except the experimental unit was grazing rotation number (*n* = 4).

Sward morphology and animal data from the first winter and grazing season were statistically analysed using the MIXED procedure of SAS. The experimental unit was the grazing group (*n* = 3). The statistical model contained fixed effects for genotype, PGSH and their interactions. Differences between means were tested for significance using the PDIFF statement and adjusted by Tukey, as appropriate. Animal data pertaining to the indoor finishing period and post-slaughter characteristics were analysed using the MIXED procedure of SAS. The experimental unit was the sub-group (finishing diet) within the previous grazing group (*n* = 3). The statistical model contained genotype, PGSH, finishing diet and their interactions as fixed effects, and the interaction between grazing group, genotype and PGSH as a random effect. Differences between means were tested for significance using the PDIFF statement and adjusted by Tukey, as appropriate.

All data pertaining to grazing behaviour measurements were analysed for each of the two consecutive 24-h measurement periods using the MIXED procedure of SAS. The experimental unit was the grazing group (*n* = 3). The statistical model contained genotype, PGSH and their interactions as fixed effects. Differences between means were tested for significance using the PDIFF statement and adjusted by Tukey, as appropriate. The data were considered statistically significant when *p* < 0.05 and considered a tendency towards statistical significance when between *p* ≥ 0.05 and *p* ≤ 0.10.

## 3. Results

### 3.1. Sward Characterisation

There was no effect of genotype or genotype × PGSH interactions for sward characteristics, morphology or feed allowance (Table 1). By design, pre-grazing herbage mass (measured above 4 cm only) and sward height were similar between PGSH treatments. Post-grazing herbage mass and PGSH were greater (*p* < 0.001) for PGSH-6 than PGSH-4. Reduced herbage mass and grazing utilisation were greater (*p* < 0.001), and canopy density tended to be greater (*p* = 0.06) for PGSH-4 than PGSH-6. Daily herbage allowance measured above 4 cm only was greater (*p* < 0.01) for PGSH-6 than PGSH-4. Consequently, PGSH-6 had a greater (*p* < 0.001) daily area grazed but lower (*p* < 0.001) residency time than PGSH-4. Stem and leaf proportions were unaffected by PGSH, but PGSH-6 had a lower (*p* < 0.05) dead proportion than PGSH-4; however, because of the greater (*p* < 0.001) herbage mass above the assigned grazing horizon, PGSH-4 had a greater (*p* < 0.001) pre-grazing leaf, stem and dead mass than PGSH-6.

### 3.2. Chemical Composition

There was no effect of genotype or genotype × PGSH interactions for grazed herbage DM, chemical composition and in vitro digestibility (Table 2). Compared to PGSH-4, PGSH-6 had greater (*p* < 0.01) DM and crude ash concentrations and tended to have a greater OMD (*p* = 0.10), DMD (*p* = 0.07) and CP concentration (*p* = 0.10). Concentrations of NDF, ADF and WSC did not differ between PGSH treatments.

### 3.3. Grazing Behaviour and Herbage Intake

During the grazing behaviour measurement period, there was no effect of genotype or genotype × PGSH interactions for DMI, eating time per kg DMI, DMI per graze bout, bite mass and intake rate (Table 3). Steer DMI (*p* < 0.01), DMI per graze bout (*p* < 0.01), bite mass (*p* < 0.05) and intake rate (*p* < 0.01) were greater, and eating time per kg DMI was lower (*p* < 0.01) for PGSH-6 compared to PGSH-4.

There was no effect of genotype or genotype × PGSH interactions for any grazing or ruminating behaviour variables (Table 4). Over a 48-h allocation, PGSH-6 tended to have greater grazing bites per day (*p* = 0.06), bite rate (*p* = 0.07) and had a greater ruminating mastication rate (*p* < 0.05) compared to PGSH-4 (Table S1). During the first 24 h of a 48-h pasture allocation, the only effect of PGSH was on ruminating mastication rate, which was greater (*p* < 0.05) for PGSH-6 than PGSH-4. However, during the final 24 h PGSH-6 had a greater eating (*p* < 0.01) and pre-hension (*p* < 0.01) time, grazing bout duration (*p* < 0.05), number of grazing bites (*p* < 0.001), bite rate (*p* < 0.01), ruminating mastication rate (*p* < 0.01), ruminating boli per ruminating bout (*p* < 0.05), ruminating bout duration (*p* = 0.09), ruminating mastications (*p* = 0.07) and ruminating mastications per bolus (*p* = 0.09).

### 3.4. Animal Growth and Carcass Traits

There were no genotype × PGSH × finishing diet interactions for animal production or carcass traits (Table 5) or ultrasonic measures of body composition (Table 6). Genotype × PGSH interactions were evident for pre-slaughter live-weight (*p* < 0.05), fat score (*p* < 0.01) and subcutaneous fat ‘redness’ (“a”) (*p* < 0.01), whereby there was no difference between PGSH within EM genotype, but within LM genotype PGSH-6 was greater than PGSH-4 (see the footnote in Table 5). There was also a PGSH × genotype interaction for pre-slaughter rib fat depth, where EM was greater than LM within PGSH-4, but the genotype did not differ within PGSH-6 (see the footnote in Table 6).

At pasture, PGSH-6 steers had a greater DMI (*p* < 0.05) and ADG (*p* < 0.05) for the complete grazing season than PGSH-4 steers. During period 1 (0.96 vs. 0.84 kg, *p* < 0.05) and period 3 (0.97 vs. 0.79 kg, *p* < 0.001) ADG was greater for PGSH-6 than PGSH-4, but there was no difference between treatments during period 2 (0.91 vs. 0.98 kg, respectively) when grazing to a common PGSH. At the end of the grazing season, PGSH-6 steers were heavier (*p* < 0.001), had greater rib fat depth (*p* < 0.05), muscle depth (*p* < 0.01), chest depth (69.4 vs. 68.6 cm; *p* < 0.05) and pelvic width (54.1 vs. 52.8 cm; *p* < 0.05) than PGSH-4 steers. During the indoor finishing period, there was no difference in DMI between PGSH treatments; however, feed conversion ratio and ADG tended (*p* = 0.08) to be greater and lower, respectively, for PGSH-6 than PGSH-4. Overall ADG from the first winter to slaughter (*p* = 0.08) and carcass weight (*p* = 0.07) tended to be greater for PGSH-6 than PGSH-4 steers. No difference in kill-out proportion, conformation score, subcutaneous fat ‘yellowness’ (“b”) or any remaining pre-slaughter ultrasonic and skeletal measurements (Table S2) was observed between PGSH treatments.

Within genotypes, although EM tended to be lighter (*p* = 0.07) than LM steers at initial housing for the first winter, there was no difference between genotypes in DMI and ADG over the first winter and subsequent turnout weight; however, EM had a greater (*p* < 0.05) first winter DMI when expressed relative to live-weight. At pasture, EM had a greater DMI (*p* < 0.05) and ADG (*p* < 0.01), and tended to be heavier (*p* = 0.10) than LM at the end of the grazing season. During the grazing season, ADG was greater for EM than LM during period 1 (1.03 vs. 0.78 kg, *p* < 0.001), with no difference during period 2 (0.93 vs. 0.95 kg, respectively) or period 3 (0.90 vs. 0.86 kg, respectively). During the indoor finishing period, EM had a greater DMI (*p* < 0.001) and tended to have a greater ADG (*p* = 0.10) than LM, and within PGSH-4, EM had a greater pre-slaughter live-weight than LM, but pre-slaughter live-weight did not differ between genotype within PGSH-6. However, the kill-out proportion was lower (*p* < 0.001) for EM, and there was no difference in carcass weight between genotype. Compared to LM, EM had a poorer carcass conformation (*p* < 0.001) and greater carcass fat score (*p* < 0.001) (the aforementioned genotype × PGSH interaction was a magnitude of effect, i.e., EM were fatter than LM within both PGSH treatments), with no differences between genotype for fat lightness (“L”) and yellowness (“b”). The ultrasonic measures of fat depth were greater, and muscle depth was lower (*p* < 0.01) at all-time points for EM than LM. Regarding skeletal measurements, EM had a longer back and narrower pelvic width at all-time points (*p* < 0.05) and a greater chest depth, evident at slaughter only (72.7 vs. 71.8 cm; *p* < 0.05), compared to LM.

During the finishing period, SC had greater DMI, ADG, pre-slaughter live-weight (*p* < 0.001), and a lower feed conversion ratio (*p* < 0.05) than SO. Compared to SO, carcass weight, kill-out proportion, fat score, muscle depth (*p* < 0.001), fat depth, conformation score (*p* < 0.01) and fat redness (“a”) (*p* < 0.01) were greater for SC. Fat lightness (“L”) and yellowness (“b”) did not differ between finishing diets.

### 3.5. Herbage Production

There was no effect of genotype or genotype × PGSH interactions for herbage production (Table 7). Compared to PGSH-4, the average paddock rest period and grazing rotation cycle length were lower (*p* < 0.05) for PGSH-6; however, PGSH-6 had a greater (*p* < 0.001) number of grazing rotation cycles. Average herbage growth rate tended to be lower (*p* = 0.07) for PGSH-6 than PGSH-4, particularly during mid-season (*p* = 0.06) resulting in a lower (*p* < 0.05) total herbage accumulation for PGSH-6. However, there was no effect of PGSH on the total amount of herbage consumed/ha through grazing, but there tended (*p* = 0.08) to be less excess herbage removed/ha as silage for PGSH-6 than PGSH-4.

## 4. Discussion

Compared to lactating dairy cow systems, there is a paucity of information on the effect of PGSH on herbage production and nutritive value, and DMI, grazing behaviour and growth performance of steers within weanling-to-beef suckler systems. The aim of this study was to address this knowledge deficit in the context of evaluating suckler steer genotypes contrasting in maturity within a temperate, rotationally stocked, grass-based production system. In terms of production systems and beef markets, consumer demand for beef produced from pasture-based diets, and ultimately “grass-fed” beef (i.e., no concentrates), is rising as it is perceived to be healthier, animal friendly and good for the environment.

### 4.1. Post-Grazing Sward Height

This study indicates that the currently recommended PGSH (4 cm) restricts steer ADG at pasture compared to PGSH-6. The poorer individual animal growth performance on the lower PGSH agrees with the findings obtained in previous research comparing 3.5 vs. 5.0 cm PGSH with dairy-bred steers [10,11] and 4.1 vs. 5.3 cm PGSH with suckled calves [36] in Ireland. Similarly, dairy cow studies in Ireland [15,16] and New Zealand [17] comparing low (2.7–4.9 cm) and high (4.2–8.7 cm) PGSH found lower milk solids production in cows grazing the lower sward residual. In contrast, Minchin and McGee [37] detected no statistically significant difference in ADG in replacement beef heifers grazing to either 4.4 or 5.6 cm, although numerically growth rate was in favour of the higher sward residual. Reasons for this inconsistency could be due to the smaller differential between the two PGSHs investigated, a ‘less severe’ low-PGSH and the lower growth potential of the older breeding heifers employed compared to other studies [10,11].

In the current study, the superior 0.10 kg ADG of steers grazing PGSH-6 equated to an additional 21 kg live-weight gain at the end of the grazing season compared to PGSH-4. The proportionate difference in ADG between PGSH treatments in the current study (0.11) was lower than the 0.21 and 0.17 proportionate differences reported by O’Riordan et al. [11] and O’Riordan et al. [10], respectively, which were equivalent to an additional 27–33 kg live-weight gain at the end of the grazing season. The relatively lower differential in the current study can be at least partially attributed to period 2 of the grazing season when, due to atypical dry weather, both treatments grazed silage area to a common PGSH of 6 cm for 43 days. This is likely to have favoured the PGSH-4 steers. Using the animal growth trajectories observed in periods 1 and 3 and excluding period 2, the estimated difference in ADG over the entire grazing season would be 0.15 kg (proportionately 0.16) rather than the 0.10 kg (proportionately 0.11) observed. Additionally, the higher proportionate differences in ADG found by O’Riordan et al. [10] and O’Riordan et al. [11] may also be due to the fact that these studies grazed tighter (3.5 cm) than the current study (4.2 cm), which could magnify the negative effects associated with grazing lower sward residuals. Collectively, these studies indicate that grazing temperate pasture excessively tightly restricts the growth rate of yearling beef cattle by 0.13–0.15 kg/day.

The superior animal growth of PGSH-6 at pasture was associated with a greater DMI of higher (statistical tendency) OMD herbage compared to PGSH-4. Herbage DMI or grazing behaviour was not measured in previous studies evaluating PGSH for beef cattle in temperature pastures [10,11,36]; however, grazing behaviour parameter values obtained for PGSH-6 steers were similar to those reported in the literature for beef cattle grazing pasture to a similar height [38,39]. In the current study, the greater herbage DMI for PGSH-6 than PGSH-4 was mainly due to grazing behaviour differences that emerged during the last 24 h rather than the first 24 h of a 48-h allocation of fresh herbage. The differences in DMI can be largely attributed to a lower bite mass and intake rate for PGSH-4 than PGSH-6, as both parameters decrease linearly with sward depletion height [40,41]. Furthermore, the shorter eating time, lower grazing bites and bite rate for PGSH-4 during the last 24 h of the allocation may be due to sward structure increasingly becoming a limiting factor, whereby animals may not have a desire to graze for longer periods of time to select out small quantities of herbage [40]. As sward structure becomes a limiting factor under such circumstances in a rotational grazing system, animals do not increase grazing time but instead stand and wait to enter a new ‘paddock’ in the rotation, leading to reduced herbage intake [42,43]. It also appears that upon entering a new paddock, PGSH-4 steers did not change their grazing behaviour or increase their DMI to compensate for the lower herbage consumption in the previous day. Consequently, offering steers ‘new’ pasture before sward structure becomes a limiting factor can increase steer DMI and subsequent ADG at pasture.

Despite the differences in DMI, with the exception of ruminating mastication rate, PGSH did not affect ruminating behaviour over the 48-h allocation. This could be due to the similarity in herbage NDF and ADF concentrations between the PGSH treatments. The greater ruminating mastication rate for PGSH-6 could be due to the higher intake rate [44], as a significant positive, albeit low, correlation (0.25) between herbage DMI and ruminating mastication rate in grazing steers has been reported [38].

In the current study, the absence of a statistically significant effect of PGSH on herbage OMD concurs with previous research [13,15], although a decrease in OMD in high compared to low PGSH has been reported elsewhere [16,17,45]. However, the decrease observed in some of the later studies [45] may be attributed to the very wide range of PGSH employed (6.6 vs. 10.5 and 14.6 cm), which are extreme in relation to modern temperate grazing systems. Similar to the current experiment, Mayne et al. [16] did not mechanically top the pasture, but they found no difference in herbage OMD between PGSH of 4.9 and 6.0 cm determined using a sward stick; however, herbage OMD decreased as PGSH increased from 6.0 to 8.7 cm (accompanied by a higher pre-grazing herbage mass).

The absence of large effects of PGSH on herbage OMD broadly supports the hypothesis of Ganche et al. [13], that animals harvested herbage from pastures to a consistent PGSH at each rotation, i.e., the animals were consuming only the herbage that had regrown since the last rotation and thus were not grazing lower into the sward profile where older herbage had accumulated and which was likely to be of lower nutritive value. This is reinforced by the similar leaf and stem proportions in the respective grazing horizons of both PGSH treatments in the current study.

Due to the lower ADG at pasture, PGSH-4 steers tended to exhibit compensatory growth during the indoor finishing period across both genotypes and finishing diets. It is surprising there was no additional compensatory growth (i.e., treatment interaction) for PGSH-4 steers offered the higher (SC) compared to the lower (SO) plane of nutrition during the finishing period. However, the compensatory index was only 0.42, which was insufficient to negate the extra weight gain achieved by PGSH-6 during the grazing season and consequently, PGSH-6 tended to have an 11 kg heavier carcass weight irrespective of genotype and finishing diet. Similarly, O’Riordan et al. [11] and Minchin et al. [36] reported that for growing beef cattle, differences in live-weight at the end of the grazing season in favour of the higher PGSH were still evident immediately post-housing, implying that ‘gut-fill’ was not a primary contributory factor.

Although there was no difference in carcass fat score between the PGSH treatments for EM genotype, LM PGSH-6 were almost 1-unit fatter than LM PGSH-4 (reflected in the statistical interaction), implying that, in addition to growth advantages, more lax grazing could be particularly beneficial for finishing LM genotypes in grass-based systems. However, there is no published literature with which to compare these findings, and this requires further research. Alternatively, if steers were not slaughtered at a similar time point and were drafted for slaughter based on carcass weight, PGSH-4 steers would require an additional 19.7 and 19.5 days on SO on SC, respectively, to obtain the same carcass weight as PGSH-6. Reducing the slaughter age of cattle can have substantial effects on lowering greenhouse gas emissions from beef production systems [46].

### 4.2. Genotype

In the current study, during the grazing season and indoor finishing period, EM had a higher DMI and ADG than LM. Although there was no statistically significant difference in grazing and ruminating behaviour parameters between the genotypes, key parameters that determine grazed herbage DMI (bite rate, intake rate and bite mass) were all numerically higher for EM than LM steers. There is relatively little published information evaluating the DMI of EM and LM beef cattle genotypes grazing pasture, and those are equivocal with higher [47] and lower [48] pasture DMI reported for EM compared to LM. Likewise, when offered a pre-dominantly forage diet supplemented with concentrates indoors, higher [49] and similar [4] DMI was reported for EM compared to LM. In terms of growth performance, most previous research has found EM to have a lower [48,50,51] ADG than LM at pasture, but not always [4], whilst the ADG differences reported during the indoor finishing period are more inconsistent, with a lower [23,50], similar [52,53] or higher [4,54] ADG reported for EM than LM, respectively. In the current study, it is important to note that the progeny of the two sire genotypes were all from LM dams. Additionally, this discrepancy across studies may be attributed to breed genetic selection [23]. Nonetheless, in this study, the higher DMI for EM at pasture and during the indoor finishing period (on SO and SC) underpins the higher ADG for EM than LM. The feed conversion ratio (based on live-weight) did not differ between genotypes at any time point in this study; however, due to the lower kill-out proportion for EM than LM [4,49,55], EM had inferior feed efficiency when expressed as carcass gain. As the higher forage DMI of EM did not yield additional carcass gain compared to LM and this resulted in a 4% lower stocking rate for EM than LM at pasture.

An objective of this study was to identify the optimum genotype for a grass-forage-only and conventional grass-forage plus concentrate steer production system. In this study, there was no genotype × finishing diet interactions. Although EM had an inferior feed efficiency, the findings of this study suggest that EM may be more suitable to grass-forage-only systems than LM as carcass weight was relatively similar (351 vs. 355 kg, respectively), but carcasses were fatter (7.4 vs. 6.2 units). These differences in carcass attributes between the genotypes on a grass-forage-only diet are similar to the values reported in Regan et al. [4] (314 vs. 319 kg and 8.5 vs. 6.1 for EM and LM, respectively). The lower carcass fat score for LM steers within both PGSH shows that a higher proportion of those carcasses failed to achieve a commercially-acceptable fat score (>6.0). A consequence of this can mean a lengthening in the production cycle or, in practical terms, a longer indoor finishing period or returning to pasture at the end of the ‘second’ winter for finishing. However, Herron et al. [47] reported that unfinished animals returned to grass for a ‘third’ grazing season with subsequent slaughtering at 28 months of age exhibited compensatory growth and superior weight gain and have a lower environmental footprint per kg carcass produced than those finished indoors on grass-forage-only at 24 months-of-age. However, the impact of a third grazing season on stocking rate in a weanling-to-beef system on a fixed land resource was not considered.

In the current study, when concentrates were supplemented during the finishing period (SC), all EM and LM steers exceeded the commercially-acceptable fat score (9.6 vs. 7.4, respectively). Therefore, LM may be more suitable than EM in a conventional forage plus concentrate input system, as in accord with previous studies [4,23], LM steers generally achieve a superior carcass weight (383 vs. 396 kg), conformation score (7.7 vs. 9.4 units) and feed efficiency than EM under that feeding regime.

### 4.3. Finishing Diet

Removing concentrates from the diet had a negative impact on steer growth and carcass traits, which is not unexpected due to the typically lower nutrient supply from grass silage [56]. The ADG achieved for SO in the current study (0.73 kg; silage DMD 756 g/kg DMD) was higher than reported previously for comparable cattle offered grass silage with similar digestibility Doyle et al. [57] (0.32 kg; 743 g/kg DMD), Clarke et al. [58] (0.49 kg; 745 g/kg DMD) and Regan et al. [4] (0.65 kg; 757 g/kg DMD); of note is that silage DMI varied substantially across those studies. The 37 kg lighter carcass for SO than SC is somewhat lower than the reductions in carcass weight obtained in previous studies (44–51 kg) [4,57,59] that had ‘consumed’ supplementary concentrates (3.0–3.2 kg DM /day) from the diet of finishing beef cattle offered high-digestibility grass silage (743–758 g/kg DMD) and slaughtered at ca. 24 months-of-age. Although it can be difficult to achieve an adequate carcass fat score with suckler-bred steers within a grass-forage-only system at 24 months of age (5.78 units, Doyle et al. [57]), it is possible to do so as demonstrated in this study (6.78) and in Regan et al. [4] (7.3).

### 4.4. Additional Considerations

In grass-based systems, herbage production has a positive impact on key profit drivers, such as stocking rate and LWG/ha [1,60]. In this study, although PGSH-6 increased steer LWG, it decreased herbage production compared to PGSH-4, but the differences in herbage production were relatively small (±506 kg DM/ha). The greater herbage accumulation for PGSH-4 agrees with the herbage regrowth principles summarised in Chapman et al. [14]. Furthermore, the longer regrowth interval [61] and a tendency for the higher sward density for PGSH-4 than PGSH-6 also contributes to a higher herbage accumulation for PGSH-4. The lower sward density for PGSH-6 could be attributed to the decreased tiller numbers as a result of lower light penetration to the base of the sward [62].

As a result of lower accumulation and greater daily demand for herbage, PGSH-6 used a 15% greater grazing area per rotation than PGSH-4, which is consistent with the literature [9,13]. In practice, this can either reduce the quantity of excess herbage removed as silage or reduce the animal stocking rate. Silage preserved per animal unit in this study was 135 kg DM (proportionately 0.07) lower for PGSH-6 than PGSH-4, resulting in 7.5 days less feeding per animal unit (assuming a combined daily demand of 17.9 kg DM for both a weanling and finishing steer when assuming an edible silage recovery of 0.78 (field to fed losses) as outlined in Keating and O’Kiely [35]). The extra 7.5 days of indoor feed supply for PGSH-4 could lead to an additional 3.2 kg carcass gain in a SO system. Minchin and McGee [37] also reported a lower silage supply (147 kg DM/heifer) for 5.6 than 4.4 cm PGSH from a one cut-silage system.

Although the stocking rate is identified as a key profit driver in temperate pasture-based beef systems [1], relatively speaking, most Irish commercial beef farms [63] are under-stocked (1.6 LU/ha) compared to research beef farms (2.6 LU/ha) [63] and commercial dairy [64] (2.1 LU/ha) farms [1]. Consequently, the 13% lower stocking rate on the grazing area (not the whole farmlet) for PGSH-6 compared to PGSH-4 is less of an issue for commercial beef systems.

The results indicate that LWG/ha was similar (2% lower for PGSH-6) for both PGSH treatments. The effect of PGSH on animal output/ha remains unclear in the literature; for example, dairy cow studies on temperate pastures reported an increase [65] (3.6 vs. 4.0 vs. 4.5 cm) and decrease [16] (5 vs. 6 cm) in milk production/ha as PGSH decreased.

Regarding genotype, the environmental footprint must be considered as outlined by Herron et al. [47], who reported similar animal production-related results to the current study. They observed that EM steers had a lower environmental footprint than LM when expressed on an LWG basis; however, the opposite occurred when expressed per kg of meat weight gain, due the superior kill-out and carcass lean proportion for LM.

Regarding the finishing diet, from an environmental perspective, it must be recognised that although concentrate supplementation increases emissions per animal, it reduces emissions per kg meat produced [47]. Therefore, strategic concentrate supplementation is often advised during the indoor finishing period where steers are slaughtered at 24 months of age [47].

Of the total steer LWG achieved from the start of the first winter to slaughter, proportionate LWGs achieved during the first winter, grazing season and indoor finishing period were 0.15, 0.57 and 0.28, respectively, in the grass-forage-only system. Corresponding values for the grass-forage + concentrate system were 0.15, 0.50 and 0.35, which is similar to Drennan and McGee [2]. This illustrates the importance of pasture management to maximise steer growth at pasture whilst producing sufficient quantities of high digestibility silage (>750 g/kg DMD) to reach carcass specification during the finishing winter.

## 5. Conclusions

Although grazing guidelines derived from dairy cow-based studies recommend grazing at a PGSH of 4 cm [12,13,14], under the conditions of this study, grazing to a PGSH of 6 cm increased steer ADG at pasture and subsequent carcass weight and, therefore, can act as an effective management strategy to reduce reliance on concentrates to improve steer ADG. However, grazing to 6 cm increases the requirement for more grazing area, which negatively impacts silage yield or stocking rate.

Although LWG was greater for EM than LM steers (both out of LM dams), there was no difference in carcass weight between the two genotypes, with little difference in the output/ha across the grazing system. It is suggested that EM steers may be more suitable than LM steers for a grass-forage-only production system due to their greater propensity for fat deposition, whereas LM steers may be more suitable for a forage and concentrate system due to a greater potential carcass value and superior feed efficiency.

Consuming concentrates during the finishing period enhanced ADG and carcass traits of steers. In the context of a grass-forage-only system, the majority (80%) of EM and LM steers in this study achieved a commercially acceptable carcass fat score (>6.0: scale 1–15) slaughtered at a relatively young age (24 months).

## Figures and Tables

**Table 1:** Effect of genotype (G-early-(EM) or late-maturing (LM)) and post-grazing sward height (PGSH-4 or 6 cm) on sward structural characteristics, sward morphology and feed allowance.

Variable	Genotype		PGSH		SEM	*p*-Value	
	EM	LM	4	6		G	PGSH
Structural characteristics							
Pre-grazing herbage mass (kg DM/ha) ^1,4^	1956	1968	2009	1931	66.7	NS	NS
Pre-grazing height (cm) ^1^	11.4	11.5	11.6	11.3	0.28	NS	NS
Canopy density (kg DM/cm/ha) ^2^	286	277	290	276	5.2	NS	0.06
Post-grazing sward height (cm) ^1^	5.1	5.2	4.2	5.8	0.06	NS	***
Post-grazing herbage mass (kg DM/ha) ^1,5^	438	445	222	589	17.2	NS	***
Reduced herbage mass (kg DM/ha) ^1,6^	1518	1523	1787	1342	64.8	NS	***
Grazing utilisation (%)	76	76	88	68	0.9	NS	***
Sward morphology ^3^							
Leaf (%)	72	72	71	73	1.5	NS	NS
Stem (%)	17	17	17	17	1.2	NS	NS
Dead (%)	11	11	12	10	0.9	NS	*
Pre-grazing leaf mass (kg DM/ha)	1340	1336	1547	1128	28.1	NS	***
Pre-grazing stem mass (kg DM/ha)	322	307	375	255	20.6	NS	***
Pre-grazing dead mass (kg DM/ha)	203	220	268	155	18.1	NS	***
Feed allowance							
Daily herbage allowance (kg DM/animal/day) ^1,7^	7.9	7.7	6.3	8.8	0.51	NS	**
Daily area grazed (m^2^/steer/day)	38	36	31	41	1.9	NS	***
Average residency time (days)	3.8	4.0	4.6	3.4	0.16	NS	***
Average weekly pasture cover (kg DM/ha) ^1^	1026	1039	1050	1015	35.6	NS	NS
Average silage herbage mass (kg DM/ha) ^1^	2292	2314	2212	2468	90.4	NS	0.06

SEM = standard error of the mean, * *p* < 0.05, ** *p* < 0.01, *** *p* < 0.001, NS = not significant. ^1^ Measured by the platemeter from 4 cm up. ^2^ Measured from the height of the assigned PGSH (4 or 6 cm). ^3^ All sward morphology parameters are measured from the height of the assigned PGSH (4 or 6 cm). ^4^ Pre-grazing herbage mass as measured by the mower (kg DM/ha) (cut from the height of the assigned PGSH (4 or 6 cm)) was 1821, 1779, 2179 and 1539 for EM, LM, 4 cm and 6 cm, respectively. ^5^ Post-grazing herbage mass as measured by the mower (kg DM/ha) (cut from the height of the assigned PGSH (4 or 6 cm)) was 256, 282, 380 and 194 for EM, LM, 4 cm and 6 cm, respectively. ^6^ Reduced herbage mass as measured by the mower (kg DM/ha) (cut from the height of the assigned PGSH (4 or 6 cm)) was 1565, 1498, 1791 and 1344 for EM, LM, 4 cm and 6 cm, respectively. ^7^ Daily herbage allowance as measured by the mower (kg DM/animal/day) (cut from the height of the assigned PGSH (4 or 6 cm)) was 6.9, 6.5, 6.7 and 6.7 for EM, LM, 4 cm and 6 cm, respectively.

**Table 2:** Effect of genotype (G-early- (EM) or late-maturing (LM)) and post-grazing sward height (PGSH-4 or 6 cm) on herbage chemical composition and in vitro digestibility during the grazing season.

Variable	Genotype		PGSH		SEM	*p*-Value	
	EM	LM	4	6		G	PGSH
DM (g/kg)	214	215	222	209	3.1	NS	**
DM composition (g/kg DM)							
OMD	776	778	763	788	10.2	NS	0.1
DMD	782	781	766	795	10.4	NS	0.07
CP	155	155	146	162	6.57	NS	0.1
NDF	423	432	429	426	15.1	NS	NS
ADF	241	235	240	237	6.8	NS	NS
WSC	271	268	262	275	18.3	NS	NS
Ash	109	108	118	101	4.1	NS	**

DM = dry matter, OMD = in vitro organic matter digestibility, CP = crude protein, NDF = neutral detergent fibre, ADF = acid detergent fibre, WSC = water soluble carbohydrates, Ash = crude ash, SEM = standard error of the mean, ** *p* < 0.01, NS = not significant.

**Table 3:** Effect of genotype (G-early- (EM) or late-maturing (LM)) and post-grazing sward height (PGSH-4 or 6 cm) on herbage dry matter intake (DMI), bite mass and intake rate during a 48-h allocation.

Variable	Genotype		PGSH		SEM	*p*-Value	
	EM	LM	4	6		G	PGSH
Steer DMI (kg/day) ^1^	6.2	5.7	4.7	7.2	0.39	NS	**
Eating time per kg DMI (min) ^2^	87.2	97.2	108.7	75.8	5.29	NS	**
DMI per grazing bout (kg)	0.65	0.60	0.50	0.75	0.051	NS	**
Bite mass (g/DM)	0.24	0.23	0.20	0.27	0.017	NS	*
Intake rate (g/min) ^3^	14.4	13.1	11.3	16.2	1.05	NS	**

SEM = standard error of the mean, * *p* < 0.05, ** *p* < 0.01, NS = not significant. ^1^ DMI during the grazing behaviour measurement period only (not the entire grazing season). ^2^ Eating time includes eat up + eat down time on the RumiWatch system. ^3^ Intake rate is calculated as (DMI × 1000)/pre-hension time).

**Table 4:** Effect of genotype (G-early-(EM) or late-maturing (LM)) and post-grazing sward height (PGSH-4 or 6 cm) on grazing and ruminating behaviour from the RumiWatch system output during the first and last 24 h of a 48-h allocation.

Variable	Genotype		PGSH		SEM	*p*-Value	
	EM	LM	4	6		G	PGSH
First 24 h							
Grazing behaviour							
Eating time (mins/d) ^1^	579	612	607	585	15.7	NS	NS
Pre-hension time (mins/d) ^2^	489	515	508	496	19.4	NS	NS
Grazing bouts (n/d)	10.3	9.3	9.7	10.0	0.50	NS	NS
Grazing bout duration (min/bout)	61.1	66.6	66.0	61.7	2.42	NS	NS
Grazing bites (n/d)	29,929	30,381	30,606	29,704	1291.7	NS	NS
Bite rate (bites/min) ^3^	61.2	59.0	60.3	59.9	0.95	NS	NS
Ruminating behaviour							
Ruminating time (min/d)	448	441	433	456	21.6	NS	NS
Ruminating bouts (n/d)	13.0	13.2	13.0	13.1	0.51	NS	NS
Ruminating bout duration (min/bout)	36.8	35.8	36.0	36.6	1.44	NS	NS
Ruminating mastications (n/d)	30,648	30,352	29,113	31,887	1756.9	NS	NS
Ruminating mastication rate (chews/min)	68.2	68.7	67.1	69.8	0.74	NS	*
Ruminating boli (n/d)	509	492	485	515	24.3	NS	NS
Ruminating mastictions per bolus (n/bolus)	58.6	60.6	58.4	60.8	1.62	NS	NS
Ruminating boli per ruminating bout (n/bout)	37.5	39.2	37.5	39.2	0.36	NS	NS
Last 24 h							
Grazing behaviour							
Eating time (mins/d) ^1^	444	453	411	486	12.1	NS	**
Pre-hension time (mins/d) ^2^	372	372	335	409	13.5	NS	**
Grazing bouts (n/d)	9.0	10.0	9.3	9.7	0.43	NS	NS
Grazing bout duration (min/bout)	52.6	51.1	48.7	55.0	1.96	NS	*
Grazing bites (n/d)	21,028	20,098	16,585	24,541	1052.0	NS	***
Bite rate (bites/min) ^3^	55.7	53.7	49.5	59.9	1.91	NS	**
Ruminating behaviour							
Ruminating time (min/d)	439	445	423	461	17.8	NS	NS
Ruminating bouts (n/d)	13.5	14.2	13.9	13.8	0.53	NS	NS
Ruminating bout duration (min/bout)	34.1	33.1	31.9	35.3	1.29	NS	0.09
Ruminating mastications (n/d)	29,238	29,792	27,392	31,638	1430.6	NS	0.07
Ruminating mastication rate (chews/min)	66.3	66.9	64.6	68.5	0.74	NS	**
Ruminating boli (n/d)	486	492	467	510	20.9	NS	NS
Ruminating mastications per bolus (n/bolus)	58.5	58.8	56.5	60.9	1.62	NS	0.09
Ruminating boli per ruminating bout (n/bout)	36.0	34.7	33.7	37.0	0.88	NS	*

SEM = standard error of the mean, * *p* < 0.05, ** *p* < 0.01, *** *p* < 0.001, NS = not significant. ^1^ Eating time includes eat up + eat down time on the RumiWatch system. ^2^ Pre-hension time only includes eat down time on the RumiWatch system. ^3^ Bite rate is calculated as (number of grazing bites/pre-hension time).

**Table 5:** The effects of genotype (G-early- (EM) or late-maturing (LM)), post-grazing sward height (PGSH-4 or 6 cm) and finishing diet (Diet-grass silage only (SO) or grass silage supplemented with 3.8 kg concentrate dry matter (SC)) on performance and carcass traits of suckler-bred steers during the first winter, grazing season and finishing period.

Variable	Genotype		PGSH		Diet		SEM		*p*-Value ^4,5,6^		
	EM	LM	4	6	SO	SC	PGSH	Diet	G	PGSH	Diet
Dry matter intake (DMI)											
First winter DMI (kg/day)	5.50	5.45	5.49	5.47	−	−	0.038	−	NS	NS	−
Pasture DMI (kg/day)	6.31	5.94	5.96	6.29	−	−	0.082	−	*	*	−
Finishing period silage DMI (kg/day)	7.84	7.32	7.63	7.52	8.49	6.66	0.070	0.079	***	NS	***
Finishing period SC DMI (kg/day)	9.75	9.23	9.54	9.44	8.49	10.49	0.070	0.079	***	NS	***
First winter DMI (g/kg live-weight)	15.7	15.1	15.4	15.3	−	−	0.14	−	*	NS	−
Pasture DMI (g/kg live-weight)	13.5	12.7	12.9	13.2	−	−	0.23	−	*	NS	−
Finishing DMI (g/kg live-weight)	15.8	15.3	15.9	15.2	14.2	16.9	0.15	0.13	*	*	***
First winter FCR ^1^	16.7	16.5	16.6	17.1	−	−	0.72	−	NS	NS	−
Pasture FCR ^1^	6.6	7.0	7.0	6.6	−	−	0.19	−	NS	NS	−
Finishing period FCR ^1^	10.6	10.9	10.4	11.2	11.6	10.1	0.29	0.33	NS	0.08	*
Average daily gain (ADG) (kg)											
First winter	0.33	0.33	0.33	0.32	−	−	0.017	−	NS	NS	−
Pasture ^2^	0.95	0.85	0.85	0.95	−	−	0.020	−	**	**	−
Finishing period	0.92	0.85	0.92	0.84	0.73	1.04	0.028	0.032	0.10	0.08	***
ADG weaning to slaughter ^3^	0.72	0.66	0.68	0.71	0.65	0.74	0.010	0.010	**	0.08	***
Carcass gain weaning to slaughter ^3^	0.40	0.38	0.38	0.40	0.36	0.42	0.006	0.006	NS	0.07	***
Live-weight (kg)											
Housing first winter	326	338	332	332	−	−	3.8	−	0.07	NS	−
Turnout to pasture	375	386	379	382	−	−	5.2	−	NS	NS	−
Housing finishing period	561	553	545	569	555	559	3.3	3.3	0.10	***	NS
Slaughter ^4^	670	653	655	669	641	682	3.9	4.5	*	*	***
Carcass traits											
Carcass weight (kg)	367	376	366	377	353	390	3.4	2.7	NS	0.07	***
Kill-out proportion (g/kg)	548	577	560	564	552	573	2.8	2.6	***	NS	***
Conformation score (1–15)	7.11	8.61	7.92	7.81	7.19	8.53	0.202	0.206	***	NS	**
Fat score (1–15) ^5^	8.50	6.81	7.58	7.72	6.78	8.53	0.146	0.162	***	NS	***
Subcutaneous fat colour											
“L”	72.3	71.9	72.3	71.9	72.3	71.9	0.29	0.31	NS	NS	NS
“a” ^6^	10.9	9.5	10.0	10.4	9.3	11.1	0.19	0.23	**	NS	**
“b”	23.3	22.8	23.0	23.2	22.8	23.4	0.24	0.24	NS	NS	NS

SEM = standard error of the mean, * *p* < 0.05, ** *p* < 0.01, *** *p* < 0.001, NS = not significant, “L” = lightness; “a” = redness; “b” = yellowness, ^1^ FCR = Feed conversion ratio (kg dry matter intake/kg live-weight gain), ^2^ ADG (kg) at pasture for 4 vs. 6 cm during period 1, period 2 and period 3 was 0.84 vs. 0.96, 0.98 vs. 0.91 and 0.79 vs. 0.97, respectively. ADG at pasture for EM vs. LM during period 1, period 2 and period 3 was 1.02 vs. 0.78, 0.93 vs. 0.95 and 0.90 vs. 0.86, respectively, ^3^ Weaning to slaughter = live-weight or carcass gain from housing during the first winter to pre-slaughter, ^4^ There was a Genotype × PGSH interaction: values of 640 vs. 667 and 670 vs. 670 for LM PGSH-4 vs. LM PGSH-6 and EM PGSH-4 vs. EM PGSH-6, respectively, SEM was 5.6, ^5^ There was a Genotype × PGSH interaction: values of 6.33 vs. 7.28 and 8.83 vs. 8.17 for LM PGSH-4 vs. LM PGSH-6 and EM PGSH-4 vs. EM PGSH-6, respectively, SEM was 0.206, ^6^ There was a Genotype × PGSH interaction: values of 8.87 vs. 10.22 and 11.18 vs. 10.58 for LM PGSH-4 vs. LM PGSH-6 and EM PGSH-4 vs. EM PGSH-6, respectively, SEM was 0.268.

**Table 6:** The effect of genotype (G-early- (EM) or late-maturing (LM)), post-grazing sward height (PGSH-4 or 6 cm) and finishing diet (Diet-grass silage only (SO) or grass silage supplemented with 3.8 kg concentrates dry matter (SC)) on ultrasonic measurements of suckler-bred steers at turnout to pasture, housing for the finishing winter and pre-slaughter.

		Genotype		PGSH		Diet		SEM		*p*-Value ^1^		
Ultrasonic Measurement	Time Interval	EM	LM	4	6	SO	SC	G + PGSH	Diet	G	PGSH	Diet
Rib fat depth	Turnout to pasture	2.54	1.86	2.25	2.16			0.073		***	NS	
	Housing finishing winter	3.77	2.45	2.79	3.43	2.99	3.22	0.174	0.205	***	*	NS
	Pre-slaughter ^1^	6.29	4.25	5.46	5.09	4.22	6.33	0.265	0.287	***	NS	**
Lumbar fat depth	Turnout to pasture	2.36	1.78	2.11	2.03			0.058		***	NS	
	Housing finishing winter	2.65	2.05	2.30	2.40	2.27	2.43	0.103	0.136	**	NS	NS
	Pre-slaughter	4.07	2.81	3.60	3.29	2.91	3.98	0.148	0.178	***	NS	**
Rump fat	Turnout to pasture	2.40	1.80	2.19	2.01			0.109		**	NS	
	Housing finishing winter	3.58	2.30	2.77	3.11	2.87	3.01	0.138	0.119	***	NS	NS
	Pre-slaughter	5.74	3.47	4.70	4.51	3.74	5.47	0.138	0.222	***	NS	**
*M. longissimus* depth	Turnout to pasture	50.1	55.5	53.6	52.0			0.75		***	NS	
	Housing finishing winter	58.4	63.0	59.0	62.4	60.3	61.1	0.68	0.64	**	**	NS
	Pre-slaughter	64.1	69.9	66.5	67.5	64.8	69.2	0.74	0.64	***	NS	***

SEM = standard error of the mean, * *p* < 0.05, ** *p* < 0.01, *** *p* < 0.001, NS = not significant, G + PGSH = genotype + post-grazing sward height. ^1^ There was a Genotype × PGSH interaction (*p* < 0.05): values of 4.03 vs. 4.47 and 6.87 vs. 5.70 for LM PGSH-4 vs. LM PGSH-6 and EM PGSH-4 vs. EM PGSH-6, respectively, SEM was 0.375.

**Table 7:** Effect of genotype (G-early- (EM) or late-maturing (LM)) and post-grazing sward height (PGSH-4 or 6 cm) on grazing rotation cycle, herbage growth rate and herbage accumulation.

Variable	Genotype		PGSH		SEM	*p*-Value	
	EM	LM	4	6		G	PGSH
Grazing rotation cycles							
Number of grazing rotation cycles	3.90	3.80	3.53	4.15	0.11	NS	***
Average rest period (days)	47.0	48.9	51.9	44.3	2.03	NS	*
Average grazing rotation cycle (days)	50.4	52.3	55.3	47.7	2.00	NS	*
Herbage growth rate (kg DM/ha/day)							
Average growth rate	41.0	40.2	42.9	38.5	1.71	NS	0.07
Early season	74.8	75.6	74.1	75.0	6.06	NS	NS
Mid-season	38.2	36.6	42.9	32.6	2.49	NS	0.06
Late season	33.5	33.3	33.1	32.8	2.53	NS	NS
Herbage accumulation (kg DM/ha)							
Total herbage accumulation	7504	7595	7802	7296	174.7	NS	*
of which grazed	4725	4605	4463	4867	438.8	NS	NS
of which removed as silage	2165	2301	2752	1714	408.8	NS	0.08
of which was the closing cover	614	689	588	715	70.8	NS	NS

SEM = standard error of the mean, * *p* < 0.05, *** *p* < 0.001, NS = not significant.

## Data Availability

The datasets used and analysed during the current study are available from the corresponding author on reasonable request.

## Supplementary Materials

The following are available online at https://www.mdpi.com/article/10.3390/ani11092623/s1, Table S1: Effect of genotype (G-early-(EM) or late-maturing (LM)) and post-grazing sward height (PGSH-4 or 6 cm) on grazing and ruminating behaviour from the RumiWatch system output during a 48-h allocation, Table S2: The effect of genotype (G-early-(EM) or late-maturing (LM)), post-grazing sward height (PGSH-4 or 6 cm) and finishing diet (FD-grass silage only (SO) or grass silage supplemented with 3.8 kg concentrates dry matter (SC)) on skeletal measurements of suckler-bred steers at turnout to pasture, housing for the finishing winter and pre-slaughter.
