# Supplementary material for: Effect of Post-Grazing Sward Height, Sire Genotype and Indoor Finishing Diet on Steer Intake, Growth and Production in Grass-Based Suckler Weanling-to-Beef Systems
Source: Animals (Basel). 2021 Sep 7;11(9):2623. doi: 10.3390/ani11092623 (PMC8470167; doi:10.3390/ani11092623)
Supplement: Supplementary file 1 [file animals-11-02623-s001.zip › animals-1341829-supplementary.pdf]

Supplementary Table S1. Effect of genotype (G - early- (EM) or late-maturing (LM)) and post-grazing sward height (PGSH - 4 or 6 cm) on grazing and ruminating behaviour from the RumiWatch system output during a 48-hour allocation.

	Genotype		PGSH		SEM	*p*-Value	
	**EM**	**LM**	**4**	**6**		**G**	**PGSH**
Grazing behaviour							
Eating time (mins/d)[1]	508	529	506	531	12.4	NS	NS
Pre-hension time (mins/d)[2]	428	441	419	449	15.2	NS	NS
Grazing bouts (n/d)	9.6	9.7	9.5	9.8	0.41	NS	NS
Grazing both duration (min/bout)	56.7	58.4	57.2	57.9	1.93	NS	NS
Grazing bites (n/d)	25317	25099	23472	26943	1101.8	NS	0.06
Bite rate (bites/min)[3]	59.0	56.9	56.0	59.9	1.32	NS	0.07
Ruminating behaviour							
Ruminating time (min/d)	441	440	425	455	18.5	NS	NS
Ruminating bouts (n/d)	13.3	13.7	13.5	13.5	0.50	NS	NS
Ruminating bout duration (min/bout)	35.4	34.4	33.9	35.9	1.31	NS	NS
Ruminating mastications (n/d)	29743	29869	28080	31532	1508.3	NS	NS
Ruminating mastication rate (chews/min)	67.2	67.8	65.9	69.1	0.78	NS	*
Ruminating boli (n/d)	494	488	473	509	20.7	NS	NS
Ruminating mastictions per bolus (n/bolus)	58.6	59.7	57.4	60.8	1.61	NS	NS
Ruminating boli per ruminating bout (n/bout)	37.3	35.8	35.3	37.8	1.02	NS	NS

SEM = standard error of the mean, * P < 0.05, NS = not significant

[1]Eating time includes eat up + eat down time on the RumiWatch system

[2]Pre-hension time only includes eat down time on the RumiWatch system

[3]Bite rate is calculated as (number of grazing bites/pre-hension time)

Supplementary Table S2. The effect of genotype (G - early- (EM) or late-maturing (LM)), post-grazing sward height (PGSH - 4 or 6 cm) and finishing diet (Diet - grass silage only (SO) or grass silage supplemented with 3.8 kg concentrates dry matter (SC)) on skeletal measurements of suckler-bred steers at turnout to pasture, housing for the finishing winter and pre-slaughter.

		**Genotype**		**PGSH**		**Diet**		**SEM**		***p-Value***		
	**Time interval**	**EM**	**LM**	**4**	**6**	**SO**	**SC**	**G+PGSH**	**Diet**	**G**	**PGSH**	**Diet**
Height at withers	Turnout to pasture	122.6	122.1	122.2	122.5			0.42		NS	NS	
	Housing finishing winter	131.3	131.2	130.8	131.7	131.4	131.1	0.68	0.70	NS	NS	NS
	Pre-slaughter	136.2	136.2	135.9	136.4	136.0	136.4	0.20	0.27	NS	NS	NS
Length of back	Turnout to pasture	121.8	120.1	120.8	121.1			0.56		0.07	NS	
	Housing finishing winter	134.5	131.9	133.1	133.3	133.2	133.2	0.60	0.61	*	NS	NS
	Pre-slaughter	139.5	137.6	138.1	138.9	138.0	139.1	0.41	0.50	*	NS	NS
Chest circumference	Turnout to pasture	170.2	171.1	171.0	170.3			0.87		NS	NS	
	Housing finishing winter	195.6	194.9	194.4	196.1	195.4	195.1	0.66	0.68	NS	0.09	NS
	Pre-slaughter	212.8	211.2	211.9	212.1	209.2	214.8	0.77	0.73	NS	NS	***
Chest depth	Turnout to pasture	61.8	61.1	61.2	61.7			0.26		NS	NS	
	Housing finishing winter	69.3	68.7	68.6	69.4	69.0	69.1	0.20	0.20	0.06	*	NS
	Pre-slaughter	72.7	71.8	72.0	72.5	71.8	72.7	0.23	0.30	*	NS	0.10
Pelvic width	Turnout to pasture	46.9	48.2	47.2	47.8			0.32		*	NS	
	Housing finishing winter	52.7	54.2	52.8	54.1	53.4	53.6	0.37	0.30	*	*	NS
	Pre-slaughter	54.6	56.0	54.9	55.7	54.7	55.9	0.33	0.35	*	NS	*

SEM = standard error of the mean, * $P < 0.05$, *** $P < 0.001$
